# (*E*)-*N*′-(2,5-Dimethoxy­benzyl­idene)-2-(8-quinol­yloxy)acetohydrazide methanol solvate

**DOI:** 10.1107/S1600536809015165

**Published:** 2009-04-30

**Authors:** Shou-Yu Wang, Liang Yuan, Liang Xu, Zhen Zhang, Yun-Peng Diao, De-Cheng Lv

**Affiliations:** a Department of Orthopaedics, The First Affiliated Hospital of Dalian Medical University, Dalian 116011, People’s Republic of China; b Department of Orthopaedics, The Second Affiliated Hospital of Dalian Medical University, Dalian 116011, People’s Republic of China; c College of Pharmacy, Liaoning University of Traditional Chinese Medicine, Dalian 116600, People’s Republic of China

## Abstract

The two mol­ecules in the asymmetric unit of the title compound, C_20_H_19_N_3_O_4_·CH_4_O, are paired *via* O—H⋯(O,N), N—H⋯O, and C—H⋯O hydrogen bonds. The mol­ecular skeleton of the acetohydrazide mol­ecule is close to planar; the benzene and quinoline mean planes form a dihedral angle of 3.9 (3)°. The crystal packing exhibits weak inter­molecular C—H⋯O hydrogen bonds and π–π inter­actions, indicated by short distances of 3.668 (3) Å, between the centroids of N-containing six-membered rings from neighbouring acetohydrazide mol­ecules.

## Related literature

For applications of 8-hydroxy­quinoline and its derivatives, see: Park *et al.* (2006 ▶); Karmakar *et al.* (2007 ▶). For a related structure, see Wen *et al.* (2005 ▶).
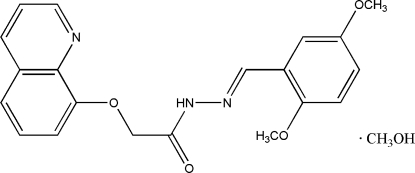


## Experimental

### 

#### Crystal data

C_20_H_19_N_3_O_4_·CH_4_O
*M* *_r_* = 397.42
Triclinic, 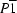

*a* = 9.4199 (12) Å
*b* = 10.8652 (14) Å
*c* = 11.1721 (14) Å
α = 93.268 (1)°
β = 112.816 (2)°
γ = 107.859 (3)°
*V* = 982.8 (2) Å^3^
*Z* = 2
Mo *K*α radiation
μ = 0.10 mm^−1^
*T* = 295 K
0.22 × 0.18 × 0.16 mm

#### Data collection

Bruker SMART CCD area-detector diffractometer
Absorption correction: multi-scan (*SADABS*; Sheldrick, 1996 ▶) *T*_min_ = 0.979, *T*_max_ = 0.985
5196 measured reflections
3456 independent reflections
2363 reflections with *I* > 2σ(*I*)
*R*_int_ = 0.019

#### Refinement

*R*[*F* ^2^ > 2σ(*F* ^2^)] = 0.050
*wR*(*F* ^2^) = 0.149
*S* = 1.03
3456 reflections
263 parameters
H-atom parameters constrained
Δρ_max_ = 0.38 e Å^−3^
Δρ_min_ = −0.33 e Å^−3^

### 

Data collection: *SMART* (Siemens, 1996 ▶); cell refinement: *SAINT* (Siemens, 1996 ▶); data reduction: *SAINT*; program(s) used to solve structure: *SHELXS97* (Sheldrick, 2008 ▶); program(s) used to refine structure: *SHELXL97* (Sheldrick, 2008 ▶); molecular graphics: *SHELXTL* (Sheldrick, 2008 ▶); software used to prepare material for publication: *SHELXTL*.

## Supplementary Material

Crystal structure: contains datablocks global, I. DOI: 10.1107/S1600536809015165/cv2552sup1.cif

Structure factors: contains datablocks I. DOI: 10.1107/S1600536809015165/cv2552Isup2.hkl

Additional supplementary materials:  crystallographic information; 3D view; checkCIF report

## Figures and Tables

**Table 1:** Hydrogen-bond geometry (Å, °)

*D*—H⋯*A*	*D*—H	H⋯*A*	*D*⋯*A*	*D*—H⋯*A*
O5—H5*A*⋯O1	0.82	2.53	2.996 (3)	117
O5—H5*A*⋯N1	0.82	2.06	2.782 (3)	147
N2—H2⋯O5	0.86	2.01	2.856 (3)	166
C12—H12⋯O5	0.93	2.51	3.305 (3)	144
C3—H3⋯O2^i^	0.93	2.60	3.220 (3)	125
C20—H20*A*⋯O2^ii^	0.96	2.59	3.511 (5)	160

Symmetry codes: (i) 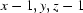; (ii) 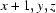.

**Fig. 1.**
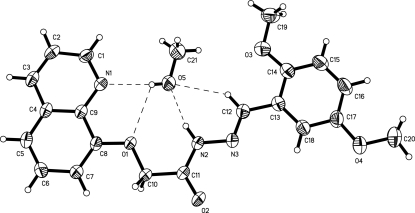
The molecular structure of (I), with displacement ellipsoids drawn at the 30% probability level. The dashed lines indicate hydrogen bonds.

## supplementary crystallographic information

### Comment

Synthesis of 8-hydroxyquinoline and its derivatives have attracted a great interest due to their interesting biological activities and applications in coordination chemistry (Park *et al.*, 2006; Karmakar *et al.*, 2007). As a part of our ongoing search for good extractants of metal ions and biologically active materials, the title compound, (I), was obtained in the reaction of quinolin-8-yloxyacetic acid hydrazide and 2,5-dimethoxybenzaldehyde.

In (I) (Fig. 1), all bond lengths and angles are normal and comparable to those in the related compound *N'*-(2-fluorobenzylidene) -2-(quinolin-8-yloxy)-acetohydrazide methanol solvate (Wen *et al.*, 2005). The mean planes of the benzene ring and the quinoline rings make a dihedral angle of 3.9 (3)°. In the crystal structure, the methanol molecule is linked to the C_20_H_19_N_3_O_4_ molecule *via* intermolecular O—H···O, N—H···O, O—H···N and C—H···O hydrogen bonds(Fig. 1 and Table 1). The crystal packing exhibits weak intermolecular C—H···O hydrogen bonds and π–π interactions proved by short distance of 3.668 (3) Å between the centroids of N-containing six-membered rings from the neighbouring molecules *L*.

### Experimental

2-(Quinolin-8-yloxy)acetohydrazide (2.18 g, 10 mmol), 2,5-dimethoxybenzaldehyde (1.66 g, 10 mmol), ethanol (40 ml) and some drops of acetic acid were added to a 100 ml flask, and refluxed for 3 h. After cooling to room temperature, the mixture was filtered. Colourless single crystals suitable for X-ray diffraction study were obtained by slow evaporation of a acetone-methanol (1:1, *v*/*v*) solution over a period of 2 d.

### Refinement

All H atoms were initially located in a difference Fourier map. C-bound H atoms were constrained to an ideal geometry, with C—H = 0.93 Å for aryl, 0.97 Å for the methylene, and 0.96 Å for the methyl H atoms, O—H = 0.82 Å and N—H = 0.86 Å. *U*_iso_(H) = 1.2*U*_eq_(C,*N*), or 1.5*U*_eq_(C) for the methyl groups, and 1.5*U*_eq_(O).

### Crystal data

**Table:**

C_20_H_19_N_3_O_4_·CH_4_O	*Z* = 2
*M**_r_* = 397.42	*F*(000) = 420
Triclinic, *P*1	*D*_x_ = 1.343 Mg m^−^^3^
Hall symbol: -P 1	Mo *K*α radiation, λ = 0.71073 Å
*a* = 9.4199 (12) Å	Cell parameters from 1903 reflections
*b* = 10.8652 (14) Å	θ = 2.5–26.9°
*c* = 11.1721 (14) Å	µ = 0.10 mm^−^^1^
α = 93.268 (1)°	*T* = 295 K
β = 112.816 (2)°	Block, colorless
γ = 107.859 (3)°	0.22 × 0.18 × 0.16 mm
*V* = 982.8 (2) Å^3^	

### Data collection

**Table:**

Bruker SMART CCD area-detector diffractometer	3456 independent reflections
Radiation source: fine-focus sealed tube	2363 reflections with *I* > 2σ(*I*)
graphite	*R*_int_ = 0.019
φ and ω scans	θ_max_ = 25.1°, θ_min_ = 2.0°
Absorption correction: multi-scan (*SADABS*; Sheldrick, 1996)	*h* = −11→10
*T*_min_ = 0.979, *T*_max_ = 0.985	*k* = −12→12
5196 measured reflections	*l* = −13→10

### Refinement

**Table:**

Refinement on *F*^2^	Primary atom site location: structure-invariant direct methods
Least-squares matrix: full	Secondary atom site location: difference Fourier map
*R*[*F*^2^ > 2σ(*F*^2^)] = 0.050	Hydrogen site location: inferred from neighbouring sites
*wR*(*F*^2^) = 0.149	H-atom parameters constrained
*S* = 1.03	*w* = 1/[σ^2^(*F*_o_^2^) + (0.0685*P*)^2^ + 0.304*P*] where *P* = (*F*_o_^2^ + 2*F*_c_^2^)/3
3456 reflections	(Δ/σ)_max_ < 0.001
263 parameters	Δρ_max_ = 0.38 e Å^−^^3^
0 restraints	Δρ_min_ = −0.33 e Å^−^^3^

### Special details

**Table:**

Geometry. All e.s.d.'s (except the e.s.d. in the dihedral angle between two l.s. planes) are estimated using the full covariance matrix. The cell e.s.d.'s are taken into account individually in the estimation of e.s.d.'s in distances, angles and torsion angles; correlations between e.s.d.'s in cell parameters are only used when they are defined by crystal symmetry. An approximate (isotropic) treatment of cell e.s.d.'s is used for estimating e.s.d.'s involving l.s. planes.
Refinement. Refinement of *F*^2^ against ALL reflections. The weighted *R*-factor *wR* and goodness of fit *S* are based on *F*^2^, conventional *R*-factors *R* are based on *F*, with *F* set to zero for negative *F*^2^. The threshold expression of *F*^2^ > σ(*F*^2^) is used only for calculating *R*-factors(gt) *etc*. and is not relevant to the choice of reflections for refinement. *R*-factors based on *F*^2^ are statistically about twice as large as those based on *F*, and *R*- factors based on ALL data will be even larger.

### Fractional atomic coordinates and isotropic or equivalent isotropic displacement parameters (Å^2^)

**Table:**

	*x*	*y*	*z*	*U*_iso_*/*U*_eq_	
O1	−0.24162 (17)	0.13371 (15)	0.32912 (13)	0.0535 (4)	
O2	0.0642 (2)	0.1461 (2)	0.64429 (16)	0.0858 (6)	
O3	0.4628 (2)	0.39500 (19)	0.28395 (18)	0.0788 (5)	
O4	0.8736 (2)	0.3699 (2)	0.7849 (2)	0.0949 (7)	
O5	−0.0293 (3)	0.2135 (3)	0.1843 (2)	0.1266 (11)	
H5A	−0.1081	0.2324	0.1818	0.190*	
N1	−0.3582 (2)	0.19146 (18)	0.08689 (17)	0.0528 (5)	
N2	0.0876 (2)	0.19807 (18)	0.45814 (17)	0.0560 (5)	
H2	0.0371	0.2020	0.3766	0.067*	
N3	0.2580 (2)	0.23818 (18)	0.51768 (18)	0.0558 (5)	
C1	−0.4175 (3)	0.2184 (2)	−0.0328 (2)	0.0601 (6)	
H1	−0.3428	0.2594	−0.0664	0.072*	
C2	−0.5851 (3)	0.1892 (2)	−0.1121 (2)	0.0643 (7)	
H2A	−0.6203	0.2104	−0.1956	0.077*	
C3	−0.6951 (3)	0.1293 (2)	−0.0644 (2)	0.0627 (6)	
H3	−0.8073	0.1086	−0.1154	0.075*	
C4	−0.6394 (3)	0.0981 (2)	0.0630 (2)	0.0536 (6)	
C5	−0.7479 (3)	0.0345 (3)	0.1181 (3)	0.0662 (7)	
H5	−0.8608	0.0141	0.0709	0.079*	
C6	−0.6890 (3)	0.0033 (3)	0.2383 (3)	0.0693 (7)	
H6	−0.7616	−0.0393	0.2731	0.083*	
C7	−0.5183 (3)	0.0346 (2)	0.3117 (2)	0.0575 (6)	
H7	−0.4796	0.0116	0.3941	0.069*	
C8	−0.4090 (2)	0.0981 (2)	0.2636 (2)	0.0474 (5)	
C9	−0.4680 (3)	0.1308 (2)	0.1355 (2)	0.0473 (5)	
C10	−0.1828 (3)	0.1048 (2)	0.4579 (2)	0.0543 (6)	
H10A	−0.2262	0.0101	0.4509	0.065*	
H10B	−0.2240	0.1450	0.5106	0.065*	
C11	0.0023 (3)	0.1532 (2)	0.5280 (2)	0.0549 (6)	
C12	0.3277 (3)	0.2839 (2)	0.4443 (2)	0.0603 (6)	
H12	0.2622	0.2859	0.3576	0.072*	
C13	0.5061 (3)	0.3334 (2)	0.4906 (2)	0.0568 (6)	
C14	0.5723 (3)	0.3921 (2)	0.4061 (3)	0.0625 (6)	
C15	0.7399 (4)	0.4419 (3)	0.4488 (3)	0.0800 (8)	
H15	0.7843	0.4798	0.3926	0.096*	
C16	0.8436 (4)	0.4366 (3)	0.5739 (3)	0.0842 (9)	
H16	0.9572	0.4722	0.6020	0.101*	
C17	0.7807 (3)	0.3791 (3)	0.6577 (3)	0.0701 (7)	
C18	0.6115 (3)	0.3272 (2)	0.6158 (3)	0.0637 (6)	
H18	0.5682	0.2879	0.6720	0.076*	
C19	0.5240 (4)	0.4653 (3)	0.2014 (3)	0.0892 (9)	
H19A	0.5871	0.5557	0.2460	0.134*	
H19B	0.4335	0.4616	0.1206	0.134*	
H19C	0.5931	0.4265	0.1814	0.134*	
C20	1.0484 (4)	0.4126 (4)	0.8254 (4)	0.1123 (12)	
H20A	1.0716	0.3572	0.7710	0.168*	
H20B	1.1020	0.4068	0.9164	0.168*	
H20C	1.0885	0.5023	0.8159	0.168*	
C21	0.0330 (4)	0.2797 (4)	0.1045 (3)	0.0957 (10)	
H21A	0.0494	0.3715	0.1245	0.144*	
H21B	−0.0432	0.2434	0.0133	0.144*	
H21C	0.1365	0.2705	0.1197	0.144*	

### Atomic displacement parameters (Å^2^)

**Table:**

	*U*^11^	*U*^22^	*U*^33^	*U*^12^	*U*^13^	*U*^23^
O1	0.0409 (8)	0.0744 (10)	0.0410 (8)	0.0212 (7)	0.0117 (7)	0.0201 (7)
O2	0.0538 (10)	0.1467 (18)	0.0475 (10)	0.0343 (11)	0.0108 (8)	0.0388 (11)
O3	0.0767 (12)	0.0900 (13)	0.0686 (12)	0.0228 (10)	0.0340 (10)	0.0253 (10)
O4	0.0494 (11)	0.1275 (18)	0.0953 (15)	0.0243 (11)	0.0215 (10)	0.0391 (13)
O5	0.0554 (12)	0.231 (3)	0.0757 (14)	0.0286 (15)	0.0217 (11)	0.0755 (17)
N1	0.0500 (11)	0.0604 (11)	0.0436 (10)	0.0208 (9)	0.0143 (9)	0.0138 (9)
N2	0.0412 (10)	0.0703 (12)	0.0432 (10)	0.0135 (9)	0.0089 (8)	0.0160 (9)
N3	0.0412 (10)	0.0607 (12)	0.0548 (11)	0.0126 (9)	0.0139 (9)	0.0118 (9)
C1	0.0621 (15)	0.0668 (15)	0.0468 (13)	0.0245 (12)	0.0164 (12)	0.0188 (11)
C2	0.0704 (17)	0.0686 (16)	0.0465 (13)	0.0317 (13)	0.0112 (12)	0.0177 (11)
C3	0.0519 (14)	0.0673 (15)	0.0549 (14)	0.0260 (12)	0.0052 (12)	0.0122 (12)
C4	0.0458 (12)	0.0561 (13)	0.0493 (12)	0.0219 (10)	0.0083 (10)	0.0069 (10)
C5	0.0405 (13)	0.0817 (17)	0.0658 (16)	0.0215 (12)	0.0121 (12)	0.0161 (13)
C6	0.0475 (14)	0.0895 (19)	0.0670 (16)	0.0188 (13)	0.0245 (12)	0.0199 (14)
C7	0.0506 (13)	0.0723 (16)	0.0487 (13)	0.0231 (12)	0.0186 (11)	0.0167 (11)
C8	0.0396 (12)	0.0538 (12)	0.0434 (11)	0.0181 (10)	0.0115 (10)	0.0068 (9)
C9	0.0452 (12)	0.0485 (12)	0.0431 (11)	0.0191 (10)	0.0124 (10)	0.0071 (9)
C10	0.0471 (13)	0.0720 (15)	0.0431 (12)	0.0235 (11)	0.0154 (10)	0.0214 (11)
C11	0.0467 (13)	0.0679 (15)	0.0437 (12)	0.0211 (11)	0.0117 (10)	0.0174 (11)
C12	0.0515 (14)	0.0667 (15)	0.0543 (14)	0.0162 (11)	0.0181 (12)	0.0106 (11)
C13	0.0510 (14)	0.0536 (13)	0.0624 (15)	0.0152 (11)	0.0240 (12)	0.0073 (11)
C14	0.0608 (15)	0.0559 (14)	0.0722 (16)	0.0179 (12)	0.0324 (13)	0.0094 (12)
C15	0.0690 (18)	0.088 (2)	0.094 (2)	0.0265 (15)	0.0457 (17)	0.0337 (17)
C16	0.0548 (16)	0.093 (2)	0.108 (2)	0.0191 (15)	0.0427 (17)	0.0303 (18)
C17	0.0496 (15)	0.0756 (17)	0.0789 (18)	0.0232 (13)	0.0203 (14)	0.0169 (14)
C18	0.0540 (15)	0.0660 (15)	0.0709 (16)	0.0177 (12)	0.0292 (13)	0.0139 (12)
C19	0.102 (2)	0.088 (2)	0.0793 (19)	0.0258 (18)	0.0458 (18)	0.0277 (16)
C20	0.0522 (18)	0.155 (3)	0.114 (3)	0.033 (2)	0.0210 (18)	0.041 (2)
C21	0.076 (2)	0.121 (3)	0.082 (2)	0.0229 (18)	0.0332 (17)	0.0275 (19)

### Geometric parameters (Å, °)

**Table:**

O1—C8	1.367 (2)	C7—C8	1.364 (3)
O1—C10	1.420 (2)	C7—H7	0.9300
O2—C11	1.219 (3)	C8—C9	1.430 (3)
O3—C14	1.364 (3)	C10—C11	1.504 (3)
O3—C19	1.410 (3)	C10—H10A	0.9700
O4—C17	1.378 (3)	C10—H10B	0.9700
O4—C20	1.435 (3)	C12—C13	1.456 (3)
O5—C21	1.371 (3)	C12—H12	0.9300
O5—H5A	0.8200	C13—C18	1.386 (3)
N1—C1	1.324 (3)	C13—C14	1.403 (3)
N1—C9	1.363 (3)	C14—C15	1.369 (4)
N2—C11	1.335 (3)	C15—C16	1.378 (4)
N2—N3	1.385 (2)	C15—H15	0.9300
N2—H2	0.8600	C16—C17	1.374 (4)
N3—C12	1.271 (3)	C16—H16	0.9300
C1—C2	1.398 (3)	C17—C18	1.385 (3)
C1—H1	0.9300	C18—H18	0.9300
C2—C3	1.355 (4)	C19—H19A	0.9600
C2—H2A	0.9300	C19—H19B	0.9600
C3—C4	1.414 (3)	C19—H19C	0.9600
C3—H3	0.9300	C20—H20A	0.9600
C4—C9	1.411 (3)	C20—H20B	0.9600
C4—C5	1.415 (3)	C20—H20C	0.9600
C5—C6	1.348 (3)	C21—H21A	0.9600
C5—H5	0.9300	C21—H21B	0.9600
C6—C7	1.408 (3)	C21—H21C	0.9600
C6—H6	0.9300		
			
C8—O1—C10	115.50 (17)	O2—C11—N2	124.4 (2)
C14—O3—C19	118.6 (2)	O2—C11—C10	117.6 (2)
C17—O4—C20	116.1 (2)	N2—C11—C10	117.96 (18)
C21—O5—H5A	109.5	N3—C12—C13	122.5 (2)
C1—N1—C9	117.75 (19)	N3—C12—H12	118.7
C11—N2—N3	119.67 (17)	C13—C12—H12	118.7
C11—N2—H2	120.2	C18—C13—C14	119.4 (2)
N3—N2—H2	120.2	C18—C13—C12	122.2 (2)
C12—N3—N2	114.67 (19)	C14—C13—C12	118.4 (2)
N1—C1—C2	124.2 (2)	O3—C14—C15	123.8 (2)
N1—C1—H1	117.9	O3—C14—C13	116.8 (2)
C2—C1—H1	117.9	C15—C14—C13	119.3 (3)
C3—C2—C1	118.4 (2)	C14—C15—C16	120.8 (3)
C3—C2—H2A	120.8	C14—C15—H15	119.6
C1—C2—H2A	120.8	C16—C15—H15	119.6
C2—C3—C4	120.1 (2)	C17—C16—C15	120.6 (3)
C2—C3—H3	120.0	C17—C16—H16	119.7
C4—C3—H3	120.0	C15—C16—H16	119.7
C9—C4—C3	117.5 (2)	C16—C17—O4	125.0 (2)
C9—C4—C5	119.7 (2)	C16—C17—C18	119.4 (3)
C3—C4—C5	122.8 (2)	O4—C17—C18	115.5 (2)
C6—C5—C4	120.5 (2)	C17—C18—C13	120.4 (2)
C6—C5—H5	119.7	C17—C18—H18	119.8
C4—C5—H5	119.7	C13—C18—H18	119.8
C5—C6—C7	120.5 (2)	O3—C19—H19A	109.5
C5—C6—H6	119.8	O3—C19—H19B	109.5
C7—C6—H6	119.8	H19A—C19—H19B	109.5
C8—C7—C6	121.0 (2)	O3—C19—H19C	109.5
C8—C7—H7	119.5	H19A—C19—H19C	109.5
C6—C7—H7	119.5	H19B—C19—H19C	109.5
C7—C8—O1	124.68 (19)	O4—C20—H20A	109.5
C7—C8—C9	119.8 (2)	O4—C20—H20B	109.5
O1—C8—C9	115.50 (18)	H20A—C20—H20B	109.5
N1—C9—C4	122.07 (19)	O4—C20—H20C	109.5
N1—C9—C8	119.45 (18)	H20A—C20—H20C	109.5
C4—C9—C8	118.5 (2)	H20B—C20—H20C	109.5
O1—C10—C11	113.06 (18)	O5—C21—H21A	109.5
O1—C10—H10A	109.0	O5—C21—H21B	109.5
C11—C10—H10A	109.0	H21A—C21—H21B	109.5
O1—C10—H10B	109.0	O5—C21—H21C	109.5
C11—C10—H10B	109.0	H21A—C21—H21C	109.5
H10A—C10—H10B	107.8	H21B—C21—H21C	109.5
			
C11—N2—N3—C12	−177.4 (2)	N3—N2—C11—O2	1.0 (4)
C9—N1—C1—C2	0.3 (3)	N3—N2—C11—C10	−177.22 (19)
N1—C1—C2—C3	−0.1 (4)	O1—C10—C11—O2	172.0 (2)
C1—C2—C3—C4	0.2 (4)	O1—C10—C11—N2	−9.6 (3)
C2—C3—C4—C9	−0.6 (3)	N2—N3—C12—C13	178.7 (2)
C2—C3—C4—C5	−179.4 (2)	N3—C12—C13—C18	3.6 (4)
C9—C4—C5—C6	−0.9 (4)	N3—C12—C13—C14	−174.8 (2)
C3—C4—C5—C6	177.9 (2)	C19—O3—C14—C15	−7.1 (4)
C4—C5—C6—C7	0.6 (4)	C19—O3—C14—C13	173.3 (2)
C5—C6—C7—C8	0.7 (4)	C18—C13—C14—O3	179.8 (2)
C6—C7—C8—O1	179.0 (2)	C12—C13—C14—O3	−1.8 (3)
C6—C7—C8—C9	−1.6 (3)	C18—C13—C14—C15	0.2 (4)
C10—O1—C8—C7	−2.3 (3)	C12—C13—C14—C15	178.6 (2)
C10—O1—C8—C9	178.19 (18)	O3—C14—C15—C16	179.5 (3)
C1—N1—C9—C4	−0.7 (3)	C13—C14—C15—C16	−0.9 (4)
C1—N1—C9—C8	179.0 (2)	C14—C15—C16—C17	1.1 (5)
C3—C4—C9—N1	0.8 (3)	C15—C16—C17—O4	−179.3 (3)
C5—C4—C9—N1	179.7 (2)	C15—C16—C17—C18	−0.5 (4)
C3—C4—C9—C8	−178.88 (19)	C20—O4—C17—C16	−6.5 (4)
C5—C4—C9—C8	0.0 (3)	C20—O4—C17—C18	174.7 (3)
C7—C8—C9—N1	−178.5 (2)	C16—C17—C18—C13	−0.2 (4)
O1—C8—C9—N1	1.0 (3)	O4—C17—C18—C13	178.7 (2)
C7—C8—C9—C4	1.2 (3)	C14—C13—C18—C17	0.4 (4)
O1—C8—C9—C4	−179.29 (18)	C12—C13—C18—C17	−178.0 (2)
C8—O1—C10—C11	−176.68 (18)		

### Hydrogen-bond geometry (Å, °)

**Table:**

*D*—H···*A*	*D*—H	H···*A*	*D*···*A*	*D*—H···*A*
O5—H5A···O1	0.82	2.53	2.996 (3)	117
O5—H5A···N1	0.82	2.06	2.782 (3)	147
N2—H2···O5	0.86	2.01	2.856 (3)	166
C12—H12···O5	0.93	2.51	3.305 (3)	144
C3—H3···O2^i^	0.93	2.60	3.220 (3)	125
C20—H20A···O2^ii^	0.96	2.59	3.511 (5)	160

Symmetry codes: (i) *x*−1, *y*, *z*−1; (ii) *x*+1, *y*, *z*.
